# Supplementary material for: Empirical methods for controlling false positives and estimating confidence in ChIP-Seq peaks
Source: BMC Bioinformatics. 2008 Dec 5;9:523. doi: 10.1186/1471-2105-9-523 (PMC2628906; doi:10.1186/1471-2105-9-523)
Supplement: Additional File 1 — A variety of html documents from the USeq web site detailing the available applications, their best usage, output file type descriptions, command line menus, etc. [file 1471-2105-9-523-S1.zip › USeqUserGuides/JavaDoc.zip!/JavaDoc/index-files/index-2.html]

B-Index


|  |  |  |
| --- | --- | --- |
| **Overview** \| Package \| Class \| Use \| **Tree** \| **Deprecated** \| **Index** \| **Help** | |  |
| **PREV LETTER**   **NEXT LETTER** | **FRAMES**    **NO FRAMES**     **All Classes** |  |


A B C D E F G H I J L M N O P Q R S T U V W X Z 

---


## **B**

**BackGroundOligo** - Class in trans.bkgrnd: A control oligo spotted multiple times on the array. **BackGroundOligo(String, int[][])** - Constructor for class trans.bkgrnd.BackGroundOligo: **balanceValues()** - Method in class edu.utah.seq.data.HeatMapMakerPosNeg: **balanceValues()** - Method in class trans.main.WindowBlockMakerTwoColor: **Bar2Gr** - Class in trans.misc: Utility to convert simple one graph xxx.bar files to xxx.gr files. **Bar2Gr(String[])** - Constructor for class trans.misc.Bar2Gr: **BarParser** - Class in edu.utah.seq.parsers: Class for reading and writing xxx.bar files. **BarParser()** - Constructor for class edu.utah.seq.parsers.BarParser: **BaseCount** - Class in util.bio.parsers: **BaseCount(int, char)** - Constructor for class util.bio.parsers.BaseCount: **baseNumber(char)** - Static method in class util.bio.calc.NucleicAcid: Returns 0 for a, 1 for c, 2 for g, 3 for t, -1 for everything else. **baseOverlapsWindow(int, SmoothingWindow)** - Method in class edu.utah.seq.data.HeatMapMaker: Is a given base contained within the window? Interbase coordinates so last window position is not included. **baseOverlapsWindow(int, Windows2HeatMapSgr.Window)** - Static method in class igb.util.Windows2HeatMapSgr: Is a given base contained within the window? **baseOverlapsWindow(int, Window)** - Method in class trans.main.WindowBlockMaker: Is a given base contained within the window? **BaseRead** - Class in edu.utah.seq.methylation454: **BaseRead(DataLine)** - Constructor for class edu.utah.seq.methylation454.BaseRead: **baseURL** - Static variable in class cgar.Util: **Bed2Gr** - Class in trans.misc: Converts a bed file to multiple gr files. **Bed2Gr()** - Constructor for class trans.misc.Bed2Gr: **BestWindowScoreExtractor** - Class in trans.misc: Finds the best overlapping Window for each region and prints that score with the region information. **BestWindowScoreExtractor(String[])** - Constructor for class trans.misc.BestWindowScoreExtractor: For each validation region, finds the single test window that 'best' overlaps. **Bin** - Class in trans.roc: Score collector for Sgr apps. **Bin()** - Constructor for class trans.roc.Bin: **Bin(double, double)** - Constructor for class trans.roc.Bin: **Bin** - Class in util.gen: Info and methods about a particular bin, used by the Histogram class. **Bin(double, double, Histogram)** - Constructor for class util.gen.Bin: **BinaryBooleanReaderWriter** - Class in util.bio.seq: Can read or write a compressed boolean[] by converting each 8 booleans to a unique string and assigns them to byte. **BinaryBooleanReaderWriter()** - Constructor for class util.bio.seq.BinaryBooleanReaderWriter: **BindingPeak** - Class in trans.main: Holds information about a binding peak within an interval. **BindingPeak(int, int, int, int, double)** - Constructor for class trans.main.BindingPeak: **BindingRegion** - Class in trans.anno: For holding information about a genomic interval, what genes are up, down, overlap, or contain this genomic inteval. **BindingRegion(int, double, String, int, int, int)** - Constructor for class trans.anno.BindingRegion: **BindingRegion(int, String, int, int)** - Constructor for class trans.anno.BindingRegion: **BindingRegion(Interval, int)** - Constructor for class trans.anno.BindingRegion: **BindingRegionComparator** - Class in trans.anno: **BindingRegionComparator()** - Constructor for class trans.anno.BindingRegionComparator: **BindingRegionGraphPrinter** - Class in trans.main: For printing chromosome specific .sgr files from IntervalPloter .txt picks files. **BindingRegionGraphPrinter(String[])** - Constructor for class trans.main.BindingRegionGraphPrinter: **BindingRegionsToGff3** - Class in trans.anno: Converts a Binding GenomicRegion file into GFF3 format. **BindingRegionsToGff3()** - Constructor for class trans.anno.BindingRegionsToGff3: **BindingRegionsToIntervals** - Class in trans.anno: Converts binding regions to intervals setting the binding region as the best window and adds 2kb on either side. **BindingRegionsToIntervals()** - Constructor for class trans.anno.BindingRegionsToIntervals: **binomialPValues(double, File, int[][], File)** - Static method in class util.gen.Num: Calculates p-values using a binomial probablility from built in R function. **binSgrs()** - Method in class trans.roc.MethodTester: Runs through an array of Sgr[] noting whether it falls in the positive regions or not positive regions **bioroot** - package bioroot: **bioroot.antibody** - package bioroot.antibody: **bioroot.oligo** - package bioroot.oligo: **bioroot.parsers** - package bioroot.parsers: **bioroot.plasmid** - package bioroot.plasmid: **bioroot.strain** - package bioroot.strain: **BL2Seq** - Class in util.bio.wrappers: This is a bit of a hack from the SimpleBlast, expand as needed. **BL2Seq(String)** - Constructor for class util.bio.wrappers.BL2Seq: **BL2SeqHit** - Class in util.bio.wrappers: BL2Seq result container. **BL2SeqHit()** - Constructor for class util.bio.wrappers.BL2SeqHit: **blankPositives()** - Method in class trans.roc.MethodTester: Resets the positive regions to their instantiation state. **Blast** - Class in gata.aligner: **Blast(AlignParams)** - Constructor for class gata.aligner.Blast: **Blast** - Class in util.bio.wrappers: Template to fire and fetch results from the ncbi bl2seq program. **Blast()** - Constructor for class util.bio.wrappers.Blast: **BlastCompompositeGenerator** - Class in util.bio.parsers: **BlastCompompositeGenerator(String[])** - Constructor for class util.bio.parsers.BlastCompompositeGenerator: **blastFASTAFiles(File, File)** - Method in class util.bio.wrappers.BL2Seq: Uses BLAST bl2seq program to align two sequences found in the seq files, won't work on windows? **BlastFilterResult** - Class in trans.tpmap: Container for a blast result: chromosome, numMatches, exactMatches, oneBPMismatches, startExactMatch. **BlastFilterResult(String, int, int, int, int)** - Constructor for class trans.tpmap.BlastFilterResult: **blastForOneBPRepeats(String, String, File)** - Static method in class trans.tpmap.TPMapOligoBlastFilter: This has been tweaked to find 1bp mismatches in a 25mer to a genome. **blastIt(String)** - Method in class gata.aligner.Blast: **blastIt(String, String)** - Method in class util.bio.wrappers.SimpleBlast: **BlastMatcher** - Class in bioroot: **BlastMatcher(ResultSet)** - Constructor for class bioroot.BlastMatcher: Constructor passed a ResultSet containing an id, name, and sequence; watch order! **BlastMatcher(String)** - Constructor for class bioroot.BlastMatcher: **BlastParser** - Class in util.bio.parsers: Copied from the BioJava Cookbook - Keith James. **BlastParser()** - Constructor for class util.bio.parsers.BlastParser: **BlastResult** - Class in util.bio.wrappers: Information related to a blast results. **BlastResult()** - Constructor for class util.bio.wrappers.BlastResult: **blastResultToHTML(BlastResult)** - Static method in class bioroot.Search: **Box** - Class in trans.graphics: Box for `IntervalPlotter`. **Box(double, double, double, Color)** - Constructor for class trans.graphics.Box: **BP\_3\_PRIME\_SHIFT** - Static variable in class edu.utah.seq.parsers.BarParser: **bpIntersectionSameChromosome(BindingRegion)** - Method in class trans.anno.BindingRegion: Assumes regions are on the same chromosome, and the end base is included, not interbase numbering. **bpIntersectionSameChromosome(Region)** - Method in class trans.anno.Region: Assumes regions are on the same chromosome, and the end base is included, not interbase numbering. **bpsIntersection(Coordinate)** - Method in class util.bio.annotation.Coordinate: Returns -1 for no overlap, 0 for complete overlap, or a positive int for the # bases of overlap. **breakAndSaveCelp(File, MapFeature[])** - Static method in class trans.cel.CelProcessor: Given a float[] celp file and the corresponding MapFeature[] will make a minimal **breakOutClusters()** - Method in class util.bio.cluster.HierarchicalClustering: **breakSaveFeatureArray(ArrayList, MapFeature[], File)** - Static method in class trans.cel.CelProcessor: Use this method to break and save a celp float[] file into chromosomal components in a directory named after the celpFile minus the .celp extension. **breakSaveIntensityValues(ArrayList, int[], String)** - Static method in class trans.tpmap.MapSplitter: Use this method to break a int[] array into different int[] by chromosome. **breakSaveIntensityValues(ArrayList, float[], String)** - Static method in class trans.tpmap.MapSplitter: Use this method to break a float[] array into different float[] by chromosome. **breakSaveIntensityValues(ArrayList, int[][], String)** - Static method in class trans.tpmap.MapSplitter: Use this method to break a int[][] array into different int[][] by chromosome. **breakSaveIntensityValues(ArrayList, float[][], String)** - Static method in class trans.tpmap.MapSplitter: Use this method to break a float[][] array into different float[][] by chromosome. **breakSavePositions()** - Method in class trans.tpmap.MapSplitter: **BrowseButtonAction** - Class in gata.aligner: **BrowseButtonAction(JTextField, JFrame, JFileChooser)** - Constructor for class gata.aligner.BrowseButtonAction: **bucket** - Variable in class util.amazon.S3Manager: **bucketName** - Variable in class util.amazon.S3Manager: **buildAndPrintIntrons()** - Method in class util.bio.annotation.ExportIntronicRegions: **buildCelas()** - Method in class trans.misc.ConvertBars2TPMapCel: **buildDirectoryFileGroups(String)** - Static method in class util.gen.IO: Converts a String of "grp1=/my/Dir1,grp2=/my/Dir2, etc that contains directories into a LinkedHashMap. **buildExecuteDoc(Primer3Pick[])** - Method in class util.bio.wrappers.Primer3Wrapper: **buildExon(GffFeature)** - Method in class util.bio.parsers.gff.GadFlyGffExtractor: **buildExtensionSetThreshold()** - Method in class trans.main.ScanChip: **buildExtensionSetThreshold()** - Method in class trans.main.ScanChromosomes: **buildExtensionSetThreshold()** - Method in class trans.main.ScanChromosomesCNV: **buildFileGroups(String, String)** - Static method in class util.gen.IO: Converts a String of "grp1=/my/file1,grp1=/my/old/file2,grp2=/my/file2, grp2=/my/new/file3,grp3=/my/new/dir/,/my/default etc" to a File[][] where the files have been broken by grouping into different File[]. **buildGeneGrp(Gff3Feature)** - Method in class util.bio.parsers.gff.DmelRel4Extractor: Creates a new gene group, call this last after having loaded the exons, transcripts, and translations. **buildGeneGrp(GffFeature)** - Method in class util.bio.parsers.gff.GadFlyGffExtractor: **buildGenericFeature(Gff3Feature)** - Method in class util.bio.parsers.gff.DmelRel4Extractor: **buildGenericFeature(GffFeature)** - Method in class util.bio.parsers.gff.GadFlyGffExtractor: **buildHTMLTableRows(String[], String[][], boolean)** - Static method in class bioroot.Util: Build HTML table rows. **buildSqlStatement(UserBean, String)** - Static method in class bioroot.Archive: Creates a sql statement for super users and standard users returns null for everyone else, lab specific. **buildSqlStatement(String, UserBean, DBUtil, boolean)** - Static method in class bioroot.DBUtil: Creates a sql statement given a UserBean and a boolean as to whether to add a DESC to the order by where appropriate. **buildTabTableRows(String[][])** - Static method in class bioroot.Archive: Build tab delimited table rows. **buildTPMAP()** - Method in class trans.misc.ConvertBars2TPMapCel: **buildTPMap()** - Method in class trans.tpmap.MummerMapper: Builds a tpmap file from a mummer results file **buildTranscript(GffFeature)** - Method in class util.bio.parsers.gff.GadFlyGffExtractor: **buildTransGroups()** - Method in class util.bio.parsers.gff.DmelRel4Extractor: **buildTransGrp()** - Method in class util.bio.parsers.gff.GadFlyGffExtractor: **buildTranslation(GffFeature)** - Method in class util.bio.parsers.gff.GadFlyGffExtractor: **bunzip2(File, File)** - Static method in class util.gen.IO: Uncompressed bzip2 files using the Ant implementation. **bunZip2(File, File)** - Static method in class util.gen.IO: Use to read in xxx.bz2 files directly to a String[]. **bzip2(File, File)** - Static method in class util.gen.IO: Uses Ants implementation of the bzip2 compression algorithm.

---


|  |  |  |
| --- | --- | --- |
| **Overview** \| Package \| Class \| Use \| **Tree** \| **Deprecated** \| **Index** \| **Help** | |  |
| **PREV LETTER**   **NEXT LETTER** | **FRAMES**    **NO FRAMES**     **All Classes** |  |


A B C D E F G H I J L M N O P Q R S T U V W X Z 

---
